# Optimal Selection of High-Performance Concrete for Post-Tensioned Girder Bridge Using Advanced Hybrid MCDA Method

**DOI:** 10.3390/ma14216553

**Published:** 2021-11-01

**Authors:** Elżbieta Janowska-Renkas, Przemysław Jakiel, Dariusz Fabianowski, Damian Matyjaszczyk

**Affiliations:** 1 Department of Building Materials Engineering, Faculty of Civil Engineering and Architecture, Opole University of Technology, ul. Katowicka 48, 45-061 Opole, Poland; d.fabianowski@po.edu.pl (D.F.); d.matyjaszczyk@doktorant.po.edu.pl (D.M.); 2 Department of Bridges, Geotechnics and Construction Processes, Faculty of Civil Engineering and Architecture, Opole University of Technology, ul. Katowicka 48, 45-061 Opole, Poland; p.jakiel@po.edu.pl

**Keywords:** optimal material selection, high-performance concrete, post-tensioned girder bridge, durability, hydrophilic nanosilica, hydrophobic nanosilica, silica fume, chemical admixture, hybrid MCDA

## Abstract

The selection of material solutions is a basic decision-making problem that occurs in engineering issues. It affects the entire life cycle of a building structure, its safe use, maintenance costs, and a need to meet requirements for sustainable development, including recycling. This paper aims at selection of the optimum composition of HPC designed for monolithic girder structures of post-tension bridges. For the analysis, a set of 12 new-generation concretes (HPC) was designed, made, and tested. A full-scope set of evaluation criteria was created and then the optimal alternative was selected. For this purpose, an advanced hybrid algorithm combining EA FAHP (Extent Analysis Fuzzy Analytic Hierarchy Process) and FuzzyTOPSIS (Fuzzy Technique for Order Preference by Similarity to an Ideal Solution) methods was used. The obtained results indicate a possibility for the practical application of the proposed algorithm by decision-making engineering staff. It can also be the basis for further research on application compared to other material and design solutions and, depending on the issue, different combination of aggregated methods.

## 1. Introduction

Problems with selection of material and structural solutions are one of the fundamental decision-making issues in technical fields, including bridge construction. It is a complex issue, requiring experience and interdisciplinary knowledge. Its complexity increases along with the requirements imposed on modern cement-based materials. Bridges made of prestressed concrete are an excellent example, where the quality of the embedded material is of great importance for the durability of the structure. The production of concrete is nowadays a great design and execution challenge, and the choice of the optimum material solution is a fundamental decision-making problem in engineering terms. It is crucial and affects the entire service life of a bridge, from its design, through its use, up to its demolition, and recycling.

Despite certain difficulties associated with erection, post-tensioned girder bridges have many advantages over classical reinforced concrete (RC) bridges, including the ability to transfer significant loads, achieve longer spans, reduce the height of cross-sections and span deflections, and have relatively high fatigue strength. These parameters showed a slight improvement due to the implementation of HPC with strength classes ranging from C50 to C100 and even C120 [1,2,3,4,5].

The first applications of HPC in prestressed bridges were recorded in the 1980s, e.g., the East Huntington Bridge over the Ohio River or the Tower Road Bridge in the state of Washington [6]. The term high-performance concrete refers to concrete that meets a wider range of requirements regarding its quality, affecting the increased durability of the structure, and thus it is not limited only to the compressive strength [7]. It is closely related to relevant properties of the concrete mix and hardened concrete, designed for a specific application.

The high-performance concrete, unlike the ordinary concrete, has a compact and tight microstructure and a low water-to-cement ratio (w/c ~ 0.2). Furthermore, it is a ecological material and thus not hazardous to human health [8,9,10,11,12]. A significant improvement in technical parameters has been obtained by modification of concrete composition, which is now a composite of cement, aggregate, water, chemical admixtures, including effective superplasticizers, and waste substances in the form of mineral additives showing pozzolanic and hydraulic properties, such as silica fume, fly ash, or blast furnace slag [4,10,13,14,15,16,17,18]. As the possibilities for modification of concrete microstructure are limited, nanotechnology solutions are more and more often used. In this case, the effect of SiO_2_ nano silicon dioxide used as a nanopowder is best known [19,20,21,22].

The most numerous group of publications on the subject are research papers, combined with theoretical verification, e.g., [23,24,25]. The authors mainly focus on the previously presented mechanical properties of concrete in time (e.g., strength, strain, Young’s modulus, etc.), especially depending on the decrease of the prestress force. They were mostly performed on full scale models. The obtained results allowed for the verification of guidelines related to HPC application for bridges [6,26,27].

Following a survey of literature it was found that implementation of new solutions (formulas) of HPCs to be used for post-tensioned bridges is still a topical issue. The following trends can be observed: increasing durability of new bridges and associated reduction in maintenance costs, creating lighter, more cost-effective structures, increasing span limits, and reducing a number of girders.

The character and complexity of the decision-making process in civil engineering is influenced by at least several factors. The first one is that design and execution conditions are never the same, which is characteristic for construction projects. The second one is the occurrence of many criteria, often contradictory, conditioned by factors that are difficult to measure, contractual, or which cannot be precisely defined. The need to take into account group ranks generated by appointed decision makers is also a difficulty. These problems are particularly evident with regard to issues undertaken by the authors in this paper and associated with the selection of concrete material solutions for advanced post-tensioned bridge span structures.

Until now, the choice of structural materials has most often been determined by standard requirements together with cost minimization. Nowadays, despite the increased number of evaluation criteria and the importance of selection of optimal material solutions, this process, in most cases, is still solved only on the basis of knowledge and experience of the decision-making civil engineers. The reason for this state of affairs is the lack of a flexible, systematized assessment model that allows an unambiguous qualification of materials. The issue is also not solved by methods that have emerged in recent years, which are used to evaluate the environmental impact of materials and construction elements. They may be divided into two main groups: those which apply a qualitative approach with a system of criteria and scoring using a simple mathematical algorithm, and a quantitative approach based on LCA (Life Cycle Assessment) and guidelines set forth in ISO 14040. Methods that are better known are: BREEAM (Building Research Establishment Environmental Assessment Method EcoProfile or LEED (Leadership in Energy and Environmental Design)—Giudice et al. [28] and Keysar and Pearce [29,30,31]. However, more often than not they are more oriented to the certification of buildings than the selection of proper materials, as they show a lot of limitations in the latter aspect. These methods use only general databases that do not consider a regional and national specific character [32,33,34,35,36,37]. The described models also share certain disadvantages: they prevent consideration of the decision-maker’s preferences, the range of criteria used is limited, and the application is limited to the analysis of selected materials only. It is a result of orientation to environment protection and that the procedures are not flexible enough to be adapted to specific construction issues [38].

In the authors’ opinion, implementation of multi-criteria analysis methods referred to as MCDA (Multi-Criteria Decision Analysis), in particular the sub-group of FGDM (Fuzzy Group Decision Making) methods allowing for the application of fuzzy values, is far more promising. There are few publications only on the application of this type of MCDA methods in the material analysis. These are works on a variety of topics and with a different scope of analysis. Paper [36] compares results obtained due to the application of four methods, including MAUT (Multi-Attribute Utility Theory) and AHP (Analytic Hierarchy Process), used to classify a selected set of building materials. Govindan and co-authors presented the DEMATEL aggregated method (Decision Making Trial and Evaluation Laboratory), ANP (Analytic Network Process), and TOPSIS (Technique for Order Preference by Similarity to an Ideal Solution) to evaluate a select group of building materials for their sustainability characteristics [39]. The combined methods DEMATEL and ANP were applied for the determination of weights and correlation between criteria, and the TOPSIS method for the final ranking of the group of materials. Akadiri et al. [40] applied Chang’s EA FAHP method (Extent Analysis Fuzzy Analytic Hierarchy Process) for the evaluation of materials used to erect a building. Related topics of the analysis were also the evaluation of concrete mix design methods [41] using the aggregated method (AHP and TOPSIS). The proposal for a hybrid algorithm of FAHP, FANP, and TOPSIS methods applied to issues connected with the optimization of maintenance activities at the bridge is given in the work [42]. One of the methods [6] applied less frequently is the Weighted Sum Method (WSM), which, however, does not allow for the application of fuzzy evaluation [43,44].

Similar studies on the selection of material solutions for repairs of concrete structures can be found in [45,46,47]. The authors applied basic methods, i.e., AHP and VIKOR (Vlse Kriterijumska Optimizacija kompromisno Resenje, which in Serbian means multicriteria optimization and compromise solution) and a simple gravimetric comparative method. There also very few papers dealing with issues identical to those analysed. Praveenkumar et al. [48] and Pinto et al. [49] applied the AHP method in the first case to evaluate the performance of HPC, in the second case to select the best mix type for the production of HPC. The paper [50] compares the application of OPC (Ordinary Portland Cement) and ultra-high-performance concrete, taking into account the environmental effects, using the SD (System Dynamics) method to analyse the development of the construction sector.

The analysis of the literature indicates that the methods applied do not show flexibility, which would allow for a quick implementation of the algorithm to any decision-making problem in the selection of material and design solutions. In most cases, the application of only basic methods entails limitations associated with them in terms of analysis, e.g., the possibility for free shaping of the levels of the decision tree, a number of applied criteria/sub-criteria or analysed alternatives. The presented difficulties clearly indicate the necessity to apply computational methods, which at the same time allow to systematize the decision-making process, take into account the required set of evaluation criteria, and which have a relatively simple procedure algorithm.

Being aware of the deficiency resulting from the literature review, this paper presents an original application of the MCDA methodology in the analysis of material solutions for HPC intended for spans of post-tensioned girder bridges.

## 2. Materials and Methodology

### 2.1. Purpose and Scope of Study with Procedure Algorithm

**Figure 1:**
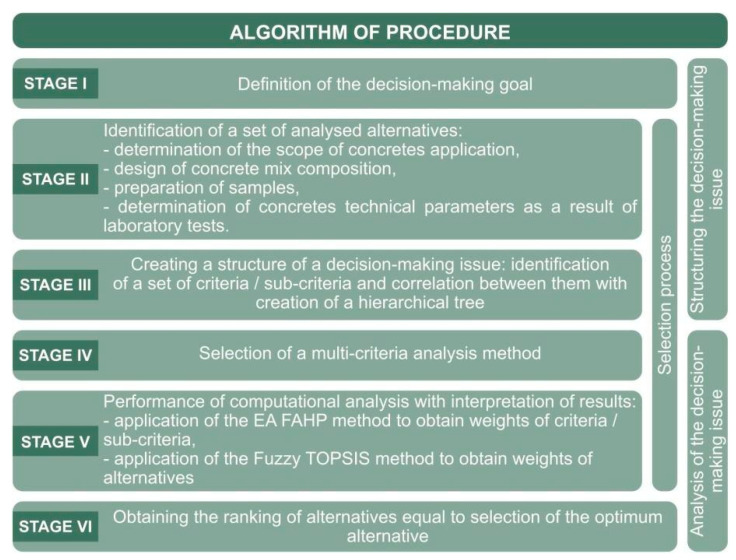
Procedure algorithm for selection of the optimum concrete mix for post-tension girder bridges.

A procedure algorithm for the proposed analysis is presented in Figure 1. Stage I defines the goal of the analysis (Section 3), which is to select the optimum composition of a concrete mix to be used in girder structures of post-tensioned girder bridges. The characteristics of the bridge adopted for the analysis is given in Section 4. The scope of the work includes, among others, the design and production of concrete specimens together with laboratory tests to determine their technical parameters necessary for evaluation. Stage II includes identification of the set of alternatives, consisting of 12 specimens with a different composition together with the results of selected laboratory tests. The next stage (Stage III) includes identification of the set of criteria together with development of a hierarchical control structure of the issue (Section 5). The fourth stage (Stage IV) presents a selection of MCDA methods to create a hybrid algorithm (EA FAHP and FuzzyTOPSIS), which has been used for the target analysis of samples (Section 6). First, a rating scale was selected for the component methods, with reference to linguistic variables, and then the evaluation process was carried out between the component elements of the hierarchical tree structure (Stage V). In the final stage (Stage VI), a ranking of the alternatives was obtained, which gave the optimal solution.

### 2.2. Presentation of the Studied Bridge

Despite the higher cost incurred during the erection of HPC post-tensioned bridges, the advantages indicated above may cause these structures to be more cost-effective than traditional ones made of reinforced concrete in the long-term operation. For that reason, a representative road viaduct over a main railway line, being a concept for a two-lane bypass of Bąków as a part of the DK-11 main road (Poland), was adopted for the analysis.

**Figure 2:**
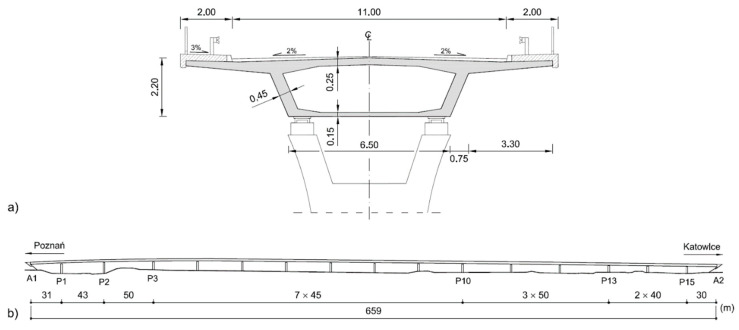
Cross-section (**a**) and side view (**b**) of high-performance concrete post-tensioned viaduct as a concept of the Bąków bypass on highway DK11 [51].

A monolithic HPC structure was adapted for that purpose, with a cross-section in the form of a post-tensioned box girder with a total width of 15.00 m and a height of 2.2 m (Figure 2a). The structure consists of 16 spans of 31 + 43 + 50 + 7 × 45 + 3 × 50 + 40 + 30 m, with a total length of 659 m (Figure 2b). The structure is equipped with inspection walkways, which together with a margin strip are 2 × 2.0 m wide, as well as with crash barriers and railing. The inclined webs of the box are 45 cm thick, the lower flange is 15 cm thick, and the deck slab is 25 cm thick.

Supports are designed as reinforced concrete structures in the form of abutments and pillars supported on direct foundations (pillars P3–P15, A2) and on two rows of piles (A1, P1, and P2). Pillars have a deep beams structure, with a separate openwork upper part in the form of inclined, short columns. Pillar heights range from 6.4 m to 11.00 m.

Properties of the concrete for the construction of monolithic post-tensioned bridges should be selected based on the following basic criteria: strength, tightness, and long-term deflections. Apart from the features mentioned, the actual execution conditions and possibilities as well as the economic criterion should also be taken into account.

Requirements for the compressive strength of concrete depend primarily on the value of internal forces that occur in the structure. Application of higher strength concrete for prestressed concrete bridges than for RC bridges results from better utilization of concrete properties in prestressed structures, as compression occurring there is transferred by the entire cross-section of concrete. In addition, concretes with higher performance parameters are less prone to deformation and therefore losses of prestress force due to the elastic shortening of the concrete also being lower. Another argument to support the use of high-performance concrete in prestressed structures is the possible safe introduction of concentrated prestress forces and anchorage of tendons. Thus, in the case of post-tensioned bridges, the concrete must have as high of a compressive strength as possible when prestressed [52]. For the same reasons, a high value of the concrete modulus of elasticity is also required, i.e., limitation of short-term (initial) losses from prestressing, concrete shrinkage and reduction of deflections of load-bearing elements. Small long-term deflections (shrinkage and creep) affect the reduction of long-term losses due to prestressing and reduction of deflections as a function of time. As in the case of reinforced concrete, the reduction of long-term deflection will be significantly affected by: reduction of the w/c ratio, use of low-shrinkage cements and selection of suitable aggregate. It should also be noted that the expectation of the high compressive strength of concrete, especially during prestressing, is in conflict with requirements to limit its shrinkage. Hence a designer has to consider each case individually [53,54].

A requirement for concrete tightness is an important parameter for any bridge structure as they are exposed to a direct impact of precipitation and large temperature fluctuations. Tight concrete can effectively protect, e.g., prestressing the steel against corrosion, which directly affects the durability and reliability of the bridge. Another important, but not the most important parameter of concrete in post-tensioned bridges, is a sufficiently high tensile strength of the concrete, as it ensures good adhesion to steel in case of elements where the post-tensioning force is transferred directly to the concrete (this applies to post-tensioned structures with adhesion). In concretes used for prestressed structures, it is also important to ensure the quality of technological processes following preparation of the concrete mix, which includes, among others, transport, pouring, compaction, and curing.

### 2.3. Materials

#### 2.3.1. Material for Testing—HPC

For the purposes of the analysed issue, 12 sets of concrete with different composition were designed and made. Design assumptions for concrete composition were focused to obtain:

- Favourable technological properties of the concrete mix and a rapid increase in the concrete early strength;
- Favourable mechanical properties, in particular high compressive strength (after 28 days);
- High durability and resistance to environmental impacts.

Such a material, in terms of its application in the building industry in the form of concrete products, should meet the requirements for its safe use in various environmental conditions, while ensuring occupational health and safety of users. These conditions are fulfilled when the concrete is designed and made of safe waste ingredients, i.e., that meet the acceptance criteria with regard to radioactivity and a content of heavy metals and hazardous compounds. These are requirements generally adopted for the raw materials used, guaranteed by manufacturers and included in their technical specifications. In addition, concrete as a product can already be tested for leachability of hazardous components. Such action is advisable and required when its production involves the content of unknown waste (sludge, rinse water of organic solvents or contaminated ash coming from other production processes). However, in case of the tests performed in this study, the ingredients used for concrete come from known suppliers and have the manufacturers’ technical data sheets, which guarantee their safe use and influence on health and environment-related factors. Therefore, the paper focuses on the determination of physical and mechanical properties of the concretes tested, which, when used in structural products, will guarantee a long service life of the structure without any repairs, as well as high durability and safety for the environment (immobilisation of compounds coming from waste in the form of silica fumes).

The economic aspect has been considered in two ways: directly, through the development of concrete sample composition, and indirectly, through evaluation of the bridge structures durability that has the impact on the environment through the extended life and reduction of maintenance and repair works.

Tests were performed for concrete mixes and hardened concretes made of Portland cement CEM I 42.5R without and with an aqueous solution of hydrophilic nanosilica (HI) and hydrophobic nanosilica colloid (HF). Nanoadditives were used in an amount of 1.5% by weight to the weight of cement. Silica fume (D) was additionally added to concretes in the amount of 8% by mass. Whereas a 40% solution of polycarboxylic superplasticizer (SP) was used as a chemical admixture in the amount of 2% by mass of cement. Consistency of concrete mixes was adjusted by the amount of water (w/c ratio from 0.23 to 0.33).

The composition of the designed concretes (S1–S12) is given in Table 1.

#### 2.3.2. Testing Methods

Properties of fresh concrete mixes and hardened concrete were tested in the accredited Construction Material Laboratory of the Faculty of Civil Engineering and Architecture at the Opole University of Technology. A scope of tests performed was adequate to satisfy the requirements for the analysed post-tensioned bridge structure. Testing included the determination of concrete mix properties in terms of: a consistency class expressed by a slump test method (with a measurement uncertainty of ±1 cm), according to PN-EN 12350-2; density (with a measurement uncertainty of 10 kg/m^3^), according to PN-EN12350-6; air content by a water column method (with a measurement uncertainty of ±0.5%), according to PN-EN12350-7.

The purpose of hardened concrete testing was to determine the early compressive strength (after 1 day of testing) and the final compressive strength (after 28 days of curing). Tests were performed according to the guidelines of PN-EN 12390-3 standard. The expanded uncertainty of measurements was ±2.9 MPa). Cubic specimens with dimensions of 100 × 100 × 100 mm were used for testing. In addition, after 28 days of concrete curing, the density was determined for cubes with the size 150 × 150 × 150 mm (for expanded uncertainty of measurement equal to ±8. 7 kg/m^3^) according to PN-EN 12390-7, water penetration depth into concrete at a pressure of 0.5 atm (expanded uncertainty ±4 mm) according to PN-EN 12390-8, water absorption (expanded uncertainty ±0.36%) and freeze-thaw resistance in water (expanded uncertainty ±0.46%), both according to PN-B-06250. The repeatability of a given test, the quantity of concrete mix, as well as dimensions and a number of cubic specimens of hardened concrete were each time in accordance with the specified standards, and the analysis of the results took into account the extended uncertainty of measurement that the test results were subject to.

#### 2.3.3. Interpretation of Results

Table 2 shows a summary of test results for the alternatives analyzed (S1–S12, Table 1), for the fresh mix (M1.1–M4.3), and for the hardened concrete (C1.1–C4.3).

Basic parameters taken into account in the evaluation of the possible application of concretes in structural elements include consistency of the concrete mix and its strength determined at the early and final stages of concrete curing period. Due to their diverse composition (Table 1), concrete mixes analysed in the study (M1.1–M4.3) have different consistency classes (denoted as ‘s’) S1–S5 (Table 2). It was shown that in the presence of hydrophilic and hydrophobic nanosilica in M3.2, M3.3, M4.2, and M4.3 mixes, there was an increase in the cone slump between 18.5 and 27.5 cm.

This caused a change in the consistency class from s1 determined for M1.1 reference mix and: M1.2, M2.1, M2.3, M3.1, and M4.1 mixes—modified by the nanoadditive content in an amount of 1.5% by mass to class:

- s2 for mixes: M1.3 (1.5% by mass of hydrophobic nanosilica—HF) and M2.2 (8% by mass of silica fume—D, and 1.5% by mass of hydrophilic nanosilica—HI);
- s4 for mixture M3.3 (1% by mass of polycarboxylate superplasticizer SP and 1.5% by mass of hydrophobic nanosilica HF);
- s5 for mixes: M3.2 (1% by mass of polycarboxylate superplasticizer SP and 1.5% by mass of hydrophilic nanosilica HI), M4.2 (8% by mass of silica fume D; 1% by mass of polycarboxylate superplasticizer SP and 1.5% by mass of hydrophilic nanosilica HI) and M4.3 (8% by mass of silica fume D; 1% by mass of polycarboxylate superplasticizer SP and 1.5% by mass of hydrophilic nanosilica HI).

The addition of 1.5% by mass of hydrophilic nanosilica HI in M1.2, as well as hydrophobic nanosilica HF in M1.3 increased the density of mixes tested compared to density of M1.1 reference mix made of CEM I 42.5R cement. Whereas in mixes containing the addition of silica fume D (M2.1), as well as silica fume D and hydrophilic nanosilica HI (M2.2) or hydrophobic nanosilica HF (M2.3), a decrease in density was observed compared to the reference mix (Table 2). On the other hand, the addition of the polycarboxylate superplasticizer SP to those mixes (M3.1, M3.2 and M3.3) increased their densities, respectively: 90 kg/m^3^ for mix M3.1, then by 100 kg/m^3^ for M3.2, and by 70 kg/m^3^ for M3.3 compared to M1.1. In addition, M4.1 mixes containing silica fume D and superplasticizer SP, as well as mixes with an additional 1.5% by mass of hydrophilic nanosilica HI (M4.2) or hydrophobic nanosilica HF (M4.3) showed an increased density compared to M1.1 reference mix.

The introduction of nanoadditives (HI and HF) to concrete mixes generally reduced the air content compared to the reference mix M1.1 made of CEM I 42.5R cement. Only M2.3 (with silica fume) and M3.3 (with silica fume, polycarboxylate superplasticizer and hydrophobic nanosilica) mixes showed an increased air content compared to the M1.1 reference mix.

It was found that the introduction of hydrophilic nanosilica HI to the concrete composition did not cause any changes in its density (in C1.2), other than under the influence of hydrophobic nanosilica HF in C1.3, where the decrease in density by about 20 kg/m^3^ was shown compared to the reference concrete (C1.1). A similar relation was also observed for the concretes: C2.1 (with 8% by mass of D) and concretes: C2.2 (with 8% by mass of D and 1.5% by mass of HI nanosilica) and C2.3 (with 8% by mass of D and 1.5% by mass of HF nanosilica). Concretes modified with superplasticizer SP (C3.1), as well as those containing 1% by mass of superplasticizer SP and 1.5% by mass of nanosilica HI (C3.2) and with 1% by mass of SP and 1.5% by mass of HF (C3.3) showed the increase in density compared to the C1.1 concrete by, respectively: 40 kg/m^3^ for C3.1, by 20 kg/m^3^ for C3.2 and by 40 kg/m^3^ for C3.3 concrete. Concretes C4.2 and C4.3, which apart from the superplasticizer and nanosilica also contained silica fume showed increased density compared to the reference concrete. On the other hand, C1.1 reference concrete and C4.1 concrete modified with 8% by mass of D fume and 1% by mass of superplasticizer SP had the same density value equal to 2390 kg/m^3^.

Simultaneous application of both nanosilica HI and HF, silica fume D, and superplasticizer SP in concretes C4.2 and C4.3, allowed for proper reduction of the w/c ratio to 0.27, which had a beneficial effect on increasing their compressive strength compared to the reference concrete C1.1. However, application of nanosilica led to a slow increase in the early strength by 180% for C4.2 concrete with HI and by 169% for C4.3 concrete with HF, respectively.

After 28 days of curing, the highest strength was demonstrated by C3.2 concrete containing polycarboxylate superplasticizer SP and hydrophilic nanosilica HI. The above indicates a compatible interaction of components used, both in terms of their quantity and type. This is also confirmed by test results of water absorption, which for C3.3 concretes (1% by mass of SP superplasticizer and 1.5% by mass of HI) was 3.9%, and for C4.2 (8% by mass of D, 1% by mass of SP and 1.5 mass % of HI) was 3%, and for C4.3 (8% by mass of D, 1% by mass of SP and 1.5 mass% of HF) was 2.6% and was considerably lower than water absorption of C1.1 reference concrete C1.1 made of cement CEM I 42.5R (5.4%). The introduction into the composition of concretes (C.2.2, C2.3) of the polycarboxylate superplasticizer in C4.2 with HI and in C4.3 with HF resulted in the best tightness among the samples tested, which was confirmed by the lowest values of their water absorption and penetration depth of water supplied under the pressure of 0.5 atm. Those concretes also showed increased resistance to frost, determined by both the lowest reduction in weight and strength compared to the reference concrete (C1.1). The samples of those concretes subject to freezing did not show any cracks or visible wastage, and the average mass loss did not exceed 5% compared to the reference samples. Those concrete types reached a F100 frost resistance level. Only in the case of concrete containing 1.5% by mass of hydrophilic nanosilica (C4.2) was the strength reduction after 100 freeze/thaw cycles greater than for the reference concrete made of pure CEM I 42.5R cement (Table 2).

Samples of those concretes subject to freezing did not show any cracks or visible mass loss, and the average mass loss did not exceed 5% compared to reference samples. Those concrete types reached a F100 frost resistance level.

The increased tightness of concretes was also confirmed by results of water absorption tests, which showed values below 5%, which, according to the requirements of the standard, allows the use of concretes with those additives in practical applications for the production of elements exposed to the direct impact of weather conditions.

### 2.4. Methodology

#### 2.4.1. Structurization of Decision Issue

**Figure 3:**
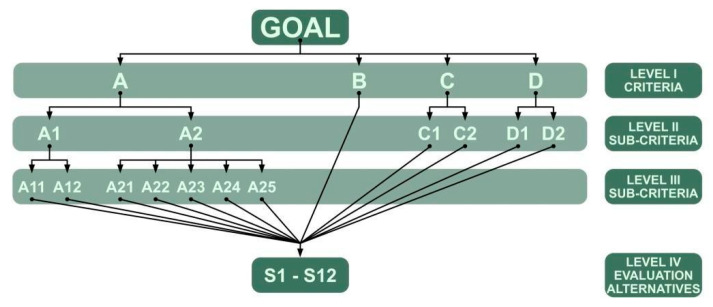
Graphical interpretation of the hierarchical control structure.

The structure of the hierarchical model includes the identification of a set of criteria that are components of the issue being analysed. The next step is to group them into thematically unified sets and to place them on appropriate levels of the hierarchical tree depending on the relations between them. A properly defined hierarchical model should enable a comprehensive assessment of the set of analysed alternatives leading to their ranking. The proposed set of criteria/sub-criteria is presented in Table 3, and the relations between them are also presented graphically in Figure 3.

The following assumptions were adopted during construction of the hierarchical tree:

- Structure safety is met a priori;
- The aggregate type and particle size distribution are identical for all alternatives being analyzed;
- Production of concrete and its transport are carried out by means of generally available equipment of concrete batching plants;
- The mix is placed in the formwork while observing the technological regime (rheology of HPCs).

During evaluation of the destimulants, the highest ranks were assigned to alternatives with the lowest values of parameters analyzed.

#### 2.4.2. Selection of Methods with Calculation Algorithm

In order to carry out the computational analysis of the issue, a hybrid algorithm consisting of two basic methods was proposed. The EA FAHP method (analysis at levels I–III of the hierarchical tree, Figure 3) was adopted to evaluate criteria with sub-criteria. The EA FAHP method has an approximate possibility to directly check the correctness of evaluation, which is not available in the case of other methods. Correctness is verified by means of a consistency ratio (CR) calculated for the modal values of the triangular fuzzy numbers constituting the ranks. This method has a certain limitation which requires the application of other basic methods. It refers to the size of the analysed matrices up to ca. 9 × 9. For larger matrices it is difficult to obtain consistency of ranking (CR < 0.1). This limitation forces the application of the FuzzyTOPSIS method for the ranking of alternatives (analysis on level IV, Figure 3). Its calculation algorithm is analogous to the classical TOPSIS, as it only requires adaptation of mathematical operations to fuzzy numbers. Analysed alternatives are compared with reference alternatives: positive-ideal and negative-ideal solutions. Determination of the ranking consists in calculation for each alternative of the distance from the reference alternatives (*d_i_^+^* and *d_i_*^−^) and in finding values of the components of the ranking vector *S_i_*. By using the hybrid method, system evaluation measures can be obtained that take into account different aspects of the issue, allowing a more precise definition of the so-called utility function, leading to identification of conditions and the analysis of their intensity.

Final evaluation of alternatives together with their ranking are obtained by aggregating the weights of the criteria/sub-criteria obtained with the EA FAHP method and then (finally) by entering them into the FuzzyTOPSIS calculation procedure. Table 4 and Table 5 present calculation algorithms of the basic methods applied with a division into individual steps. For the EA FAHP method, the original notation of the AHP method [56] was retained. Table 6 shows summary of rating scales with relevant linguistic equivalents.

## 3. Results and Discussion

Calculations in the analysed issue based on results of the design and research part (Section 4) were carried out by means of the computational procedure developed according to the algorithm presented in Section 6. Both in the case of the pairwise comparison of criteria and sub-criteria in the EA FAHP method, as well as in the ranking of analyzed alternatives S1–S12 in terms of criteria/sub-criteria in the FuzzyTOPSIS method, the evaluation process was conducted by a team of DM (decision makers). The team consisted of persons with a long-term experience in civil engineering, as well as in the technology of building and cement-based materials. Based on the ranks obtained, differences were found only in nine cases (approximately 5% of the total ranks), and the discrepancy was by 1 unit in each case. Discrepancies in the final ranks were determined by aggregation of individual judgements.

**Figure 4:**
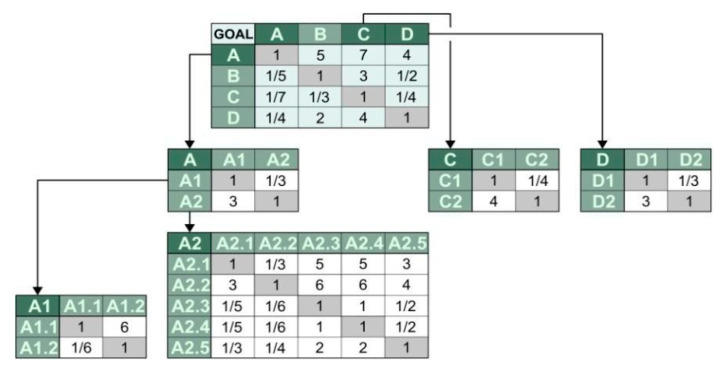
Matrices of ranking obtained in result of pairwise comparisons for main criteria and sub-criteria.

When analysing the obtained pairwise comparison results for criteria/sub-criteria using the EA FAHP method, the value of the CR coefficient was within the range 0.0000–0.0616. Matrices of ranking obtained in the result of pairwise comparisons for main criteria A–D and sub-criteria A, C, and D in EA FAHP method using TFN (triangular fuzzy number, rating is given in a form of modal values) are shown in Figure 4. It shows that criterion A (material criterion) together with sub-criterion A2 (criterion for evaluation of selected physical properties in relation to hardened concrete) are decisive in ranking the alternatives, while criterion C (economic criterion) is the least important.

In the case of the C2 criterion, ranking of alternatives was estimated based on predicted parameters of the designed structure, i.e., its target technical condition. The mean values from the measurements were adopted as ranks of the alternatives determined by features tested with laboratory methods, and the standard deviation was approximated by fuzzy ranks.

Table 7 shows the decision matrix of the FuzzyTOPSIS method with the output ranks of concrete samples and aggregated weights of criteria and subcriteria w_i_^E^ with (marked with *) obtained by means of EA FAHP method.

**Figure 5:**
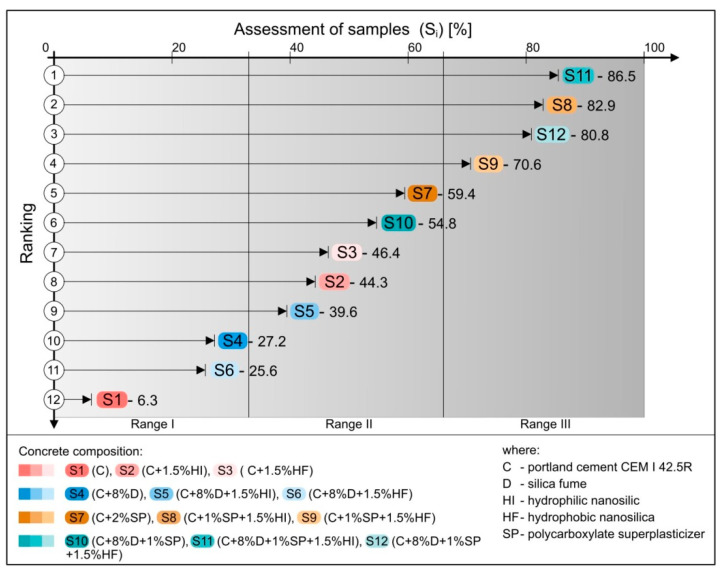
Ranking of concrete mixes tested based on the final score, taking into account physical and mechanical properties and economic and environmental aspects (suggested ranges of application are marked as Ranges I–III).

Final ranking of alternatives (Si) of selected material solutions for concretes S1–S12 is presented in Table 8 and Figure 5.

In result of the analysis performed, the highest rank was obtained by S11 sample, then concretes marked as S8 and S12, with ranks 0.865, 0.829, and 0.808, respectively (Figure 5). It can be concluded that the results for these concretes are almost equivalent and the material is preferred for the defined application. Analysing the criteria matrix, it can be stated that they were affected by the compressive strengths (early and after 28 days), and to a lesser extent by freeze-thaw resistance and workability (Figure 4).

The highest rank obtained for S11 concrete is mainly due to its very good technical parameters, confirmed by tests. The use of additives in the form of silica fume, hydrophilic nanosilica, and a polycarboxylate superplasticizer resulted in high early and final strength, reduced water absorption, and increased freeze-thaw resistance compared to other samples. Simultaneous application of nano- and microsilica has proved to be a very beneficial solution, leading to densely packed spaces around cement grains and accelerated concrete curing (observed already after one day of curing). Such an effect is caused by an additional C-S-H phase, obtained in the pozzolanic reaction of silica fume and calcium hydroxide, in the form of a gel filling the micropores. Its presence causes better sealing of the cement matrix and a visible increase in the final strength of the concrete. The technical parameters obtained also indicate its increased durability.

To make results (ranking) more clear in the context of HPC applications for post-tensioned girder bridges, three rating ranges (ranges I–III) were distinguished, i.e., 0–33%, 33–67%, and 67–100%, which can provide a convenient reference scale for designers and administrator (Figure 5). The highest range (III) indicates the preferred application of concretes in the case the investor has an unlimited budget, taking into account the highest mechanical parameters of the material. Apart from the concretes indicated above, sample S9 was also included in that range.

Concretes marked as S7, S10, S3, S2, and S5 in the ranking order are in range II and on the basis of analyses (Table 7) it can be noted that despite slightly lower mechanical properties than samples from range I, they belong to the more cost-effective solutions to a much greater extent than concretes (S4, S6, S1) from range I (Figure 5). As a result of the economic analysis of mixes (criterion C1), it was found that the cost of concretes S4, S7, and S10 was comparable to S1 reference concrete. The additives used did not affect the price increase, but significantly improved the technical parameters of the concretes. The final rank of S7 concrete that got the highest rank out of the three concretes is almost 10 times higher than the value for S1 reference concrete. Apart from the economic factor, these alternatives are an indication for the further development of HPC in bridge applications, representing solutions with greater potential in terms of sustainability.

On the basis of the performed tests and multi-criteria analysis, as well as previous experience in the use of HPCs for post-tensioned bridge structures, it can be assumed that this material also has ecological and economic potential for the projects implemented (e.g., lower consumption of concrete → lower energy consumption in production processes → lower amount of material to be disposed after the bridge life expires, higher durability → lower maintenance costs).

It should also be noted that the rational detailed specification of criteria and the use of linguistic variables in the procedure (qualitative description) allow for a clear evaluation of the analysed samples, regardless of their design composition and mechanical properties. This is favoured by the establishment of interdisciplinary teams of DMs, recommended due to the complexity of the issue.

## 4. Conclusions

Research on high-performance concretes for post-tensioned bridges, lasting for three decades, indicates the importance of the problem. At the same time, the presence of many criteria and almost unlimited design possibilities for the composition of concretes, cause the occurrence of considerable difficulties in obtaining an unambiguous solution in the selection of optimum material. Noting the lack of similar publications, this paper presents a computational and decision-making model for the evaluation and ranking of this type of concrete. The aggregated algorithm, which involves the EA FAHP and FuzzyTOPSIS methods, has been verified on a group of 12 samples of concretes designed for this purpose. On this basis, the following conclusions have been specified:

- The algorithm allows to consider various decision-making factors affecting the selection of the most favourable concrete and, ultimately, the quality of the execution and durability of post-tensioned girder bridges, taking into account economic and ecological factors;
- The results obtained confirmed the correctness of the algorithm and its application potential—it can be used as a tool supporting the development of new material solutions in bridge construction;
- The algorithm, unlike the methods used so far (single methods with the limited range of ranks to crisp values), allows for the mapping of real concrete quality assessment processes, taking into account uncertainty, incomplete information, as well as subjective and group ranks (possible introduction of their fuzzy values);
- The practical aspect of the proposed model is the possibility of its relatively free adaptation to the specific character of the problem, without limiting a number of analysed alternatives (concrete samples) and the possibility for almost free shaping of the hierarchical tree;
- Modification of concrete composition by the simultaneous combination of silica fume and polycarboxylate superplasticizer added in the presence of hydrophilic or hydrophobic nanosilica proved to be a solution that allows for a significant reduction of the water-cement ratio, which has a beneficial effect on the improvement of physical parameters of concrete mixes and mechanical properties of hardened concretes. The above solution guarantees tight placing and compacting of mixes in moulds, their proper curing, as well as obtaining increased durability of hardened concretes demonstrated by a quick increase of early and final strength, high tightness, and very good resistance to weather conditions (temperatures below zero).

It shall be also noted that the method has certain shortcomings, i.e., it is not possible to take into account a long-term dependence of the criteria ranks, e.g., associated with fluctuation of market conditions. Therefore, it seems necessary to improve the computational model by implementing the mathematical optimisation technique to support the analysis of multi-criteria decision-making problems, taking into account specific constraints and requirements. Other directions for further research should also consider the setting of precise ranges of final ranks depending on such factors as the technological, ecological, or economic aspects depending on the adopted criteria of sustainable development (balance of energy required for production, maintenance, and recycling of the structure). Despite over 30-year experience in implementation of HPC, it should be noted that there is relatively little data on the durability and recycling of this material applied for bridge structures. In the current state of knowledge, these data can only be estimated.

To sum up, the proposed hybrid MCDA method enables the identification of dominant criteria (sub-criteria) and selection of the most advantageous solution (concrete) for the application in question. The analysis is based on technical parameters of selected HPCs obtained in lab testing. The proper selection of structural material is particularly important in the case of post-tensioned bridges using HPC, as many parameters can have a decisive impact on the quality of execution, as well as the durability of the structure.

The obtained results, as the outcome of a large spectrum of decision-making processes, indicate a possibility for popularization of the methodology proposed, provided that supplementary research is done (e.g., increasing the number of experts of the DM team, diversification of disciplines or the use of specialist software helpful in the evaluation of alternatives according to selected criteria). This may improve the technical level of the execution of post-tension girder bridges made of HPC.

## Figures and Tables

**Table 1:** Composition of concretes with and without the addition of hydrophilic and hydrophobic nanosilica, as well as silica fume and polycarboxylate superplasticizer.

Identification of Concrete	Concrete Composition	w/c	Water	Aggregate	Cement
			(kg/m^3^)	(kg/m^3^)	(kg/m^3^)
S1	C	0.33	179.48	1619.59	549.68
S2	C + 1.5% HI		171.23		
S3	C + 1.5% HF		171.23		
S4	C + 8% D	0.36	192.58	644.96	467.33
S5	C + 8% D + 1.5% HI		185.57		
S6	C + 8% D + 1.5% HF		185.57		
S7	C + 2% SP	0.23	116.29	1619.59	549.68
S8	C + 1% SP + 1.5% HI		113.54		
S9	C + 1% SP + 1.5% HF		113.54		
S10	C + 8% D + 1% SP	0.27	139.12	1644.96	467.33
S11	C + 8% D + 1% SP + 1.5% HI		132.11		
S12	C + 8% D + 1% SP + 1.5% HF		132.11		

Abbreviations: **C**—CEM I 42.5R Portland cement; **D**—silica fume; **HI**—hydrophilic nanosilica; **HF**—hydrophobic nanosilica; **SP**—polycarboxylate superplasticizer.

**Table 2:** Test results of concrete mix and hardened concrete.

Alternatives	Mixture	w/c	Properties of Fresh Concrete Mixtures			Concrete	Properties of Hardened Concretes						
			Cone Slump	Density of Mix	Air Content		Concrete Density	Compressive Strength		Water Absorption	Frost Resistance		Water Permeability
								1 Day	28 Days		Weight Loss	Decrease in Compressive Strength	
			(cm)	(kg/m^3^)	(%)		(kg/m^3^)	(MPa)	(MPa)	(%)	(%)	(%)	(mm)
S1	M1.1	0.33	2.5	2340	2.7	C1.1	2390	17.9	55.2	5.4	1.9	8.5	52
S2	M1.2		4	2360	2.2	C1.2	2390	31.9	58.2	4.8	0.18	0.8	20
S3	M1.3		4.5	2350	1.9	C 1.3	2370	26.9	61.6	4.9	0.19	1	22
S4	M2.1	0.36	3	2320	2.3	C 2.1	2340	16.8	51.9	4.8	1.1	5.7	34
S5	M2.2		4.5	2330	2.6	C2.2	2350	20.4	57.0	4.9	0.2	0.4	11
S6	M2.3		3.5	2330	3.3	C 2.3	2350	15.3	54.4	4.9	0.18	0.3	10
S7	M3.1	0.23	3	2430	1.9	C 3.1	2430	48.8	65.2	4.6	0.4	6.9	16
S8	M3.2		23.0	2440	2.5	C3.2	2410	51.5	93.1	4.5	0.15	0.6	16
S9	M3.3		21.0	2410	3.7	C3.3	2430	39.4	82.8	3.9	0.15	1.8	18
S10	M4.1	0.27	3	2380	2.1	C 4.1	2390	28.1	75.2	4.2	0.6	3.2	21
S11	M4.2		30	2360	1.8	C4.2	2430	50.2	86.5	3	0.04	10.1	7
S12	M4.3		30	2360	2.0	C4.3	2410	48.1	86.3	2.6	0.08	0.2	6

**Table 3:** Classification and description of evaluation criteria.

Criterion/Sub-Criterion			Specification
**A**	**Criterion for Material**, a criterion to evaluate material properties		
	A1	For concrete mix	
		A1.1	Criterion for concrete workability (testing consistence of concrete mix by the concrete slump test method)
		A1.2	Air content in concrete mix
	A2	For hardened concrete	
		A2.1	Early strength (strength gain over time)
		A2.2	Compressive strength after 28 days
		A2.3	Water absorption
		A2.4	Water penetration depth in concrete
		A2.5	Freeze-thaw resistance
**B**	**Technological Criterion** associated with curing of concrete mix		
**C**	**Economic Criterion**		
	C1	Cost of mix production (per unit volume—per 1 m^3^)	
	C2	Cost of structure maintenance. Evaluation of service life of structural elements with evaluation of the costs of inspections and regular maintenance of the structure (e.g., de-icing)	
**D**	**Environmental Criterion**, a degree of environmental impact minimization considered comprehensively at the stage of a structure life cycle. Under the main criterion, two sub-criteria were distinguished, which determine the structure impact on the environment at the design, assembly, and service stages (life cycle of the structure together with post-demolition aspects). According to guidelines [55], the bridge service life has been assumed as a minimum of 100 years.		
	D1	Assessment of concrete degradation impact on environment (harmfulness)	
	D2	Evaluation of possible recycling/disposal of materials used at the end of the structure life cycle. Possible disposal of non-renewable materials in a manner friendly to environment, recycling, and reuse of materials originally used to produce the new ones; the higher the percentage of recyclable material and the higher concrete strength parameters, the higher the ranking.	

**Table 4:** Calculation algorithm of modified EA FAHP method, developed based on [56,57,58,59].

**Step 1.**	Development of evaluation matrix as a result of pairwise comparison of criteria/sub-criteria using TFN (triangular fuzzy number) (*l_ij_*, *m_ij_*, *u_ij_*):											
	${\tilde{A}}_{ij}=\left({\tilde{a}}_{ij}\right)=\left[\begin{array}{cc} \begin{array}{cc} {\tilde{a}}_{11}=\left(1,\;1,\;1\right) & {\tilde{a}}_{12}=\left(l_{12},\;m_{12},\;u_{12}\right)\\ {\tilde{a}}_{21}=\left(l_{21},\;m_{21},\;u_{21}\right) & {\tilde{a}}_{22}=\left(1,\;1,\;1\right) \end{array} & \begin{array}{cc} \ldots & {\tilde{a}}_{1n}=\left(l_{1n},\;m_{1n},\;u_{1n}\right)\\ \ldots & {\tilde{a}}_{2n}=\left(l_{2n},\;m_{2n},\;u_{2n}\right) \end{array}\\ \begin{array}{cc} \vdots & \vdots \\ {\tilde{a}}_{n1}=\left(l_{n1},\;m_{n1},\;u_{n1}\right) & {\tilde{a}}_{n2}=\left(l_{n2},\;m_{n2},\;u_{n2}\right) \end{array} & \begin{array}{cc} \ddots & \vdots \\ \ldots & {\tilde{a}}_{nn}=\left(1,\;1,\;1\right) \end{array} \end{array}\right]$											(1)
	where:											
	${\tilde{a}}_{ij}={\left[{\tilde{a}}_{ji}\right]}^{-1}={\left(l_{ij},\;m_{ij},\;u_{ij}\right)}^{-1}=\left(\frac{1}{u_{ij}},\;\frac{1}{m_{ij}},\frac{1}{l_{ij}}\right)$											(2)
**Step 2.**	Verification of ranking correctness by means of a CR obtained for modal values of fuzzy numbers:											
	$CR=\frac{\lambda_{max}-n}{\left(n-1\right)\times RI}$											(3)
	where:											
	*λ_max_*	—	a maximum value of the eigenvector of **A** matrix created from modal values of fuzzy numbers;									
	*n*	—	a rank of the **A** matrix (a number of criteria, sub-criteria, and objects being compared);									
	*RI*	—	a random indicator of inconsistency in assigning the ranks, dependent on the number of elements compared (*n*), developed with the use of statistical processing of results in numerous computer simulations [56] given below:									
	*n*		1	2	3	4	5	6	7	8	9	10
	*RI*		0.00	0.00	0.58	0.90	1.12	1.24	1.32	1.41	1.45	1.49
**Step 3.**	Calculation of a synthetic measurement indicator for ranks according to the corrected equation suggested by [58,59]:											
	${\tilde{S}}_{i}=\frac{\tilde{R}{\tilde{S}}_{i}}{\sum_{j=1}^{n}\tilde{R}{\tilde{S}}_{j}}=\left(\frac{\sum_{j=1}^{n}l_{ij}}{\sum_{j=1}^{n}l_{ij}+\sum_{k=1,\;k\neq i}^{n}\sum_{j=1}^{n}u_{kj}},\;\frac{\sum_{j=1}^{n}m_{ij}}{\sum_{k=1,\;k\neq i}^{n}\sum_{j=1}^{n}m_{kj}},\;\frac{\sum_{j=1}^{n}l_{ij}}{\sum_{j=1}^{n}u_{ij}+\sum_{k=1,\;k\neq i}^{n}\sum_{j=1}^{n}l_{kj}}\right)$											(4)
**Step 4.**	Calculation of components of weight vector expressed by crisp values using Yager ranking method:											
	$W_{i}=S_{i}\left({\tilde{S}}_{i}\right)=\frac{l_{i}+m_{i}+u_{i}}{3}$											(5)
	final after standardization:											
	$W_{i}=\frac{S_{i}}{\sum_{i=1}^{n}S_{i}}$											(6)

**Table 5:** Calculation algorithm of FuzzyTOPSIS method developed based on [60,61,62].

**Step 1.**	Determination of decision matrix			
	$\tilde{D}={\left[{\tilde{x}}_{ij}\right]}_{mxn}=\begin{array}{cc} & \begin{array}{ccc} c_{1\;} & c_{2\;} & \begin{array}{cc} \ldots & \;c_{n} \end{array} \end{array}\\ \begin{array}{c} A_{1}\\ A_{2}\\ \begin{array}{c} \vdots \\ A_{n} \end{array} \end{array} & \left[\begin{array}{ccc} {\tilde{x}}_{11} & {\tilde{x}}_{12} & \begin{array}{cc} \ldots & {\tilde{x}}_{1n} \end{array}\\ {\tilde{x}}_{21} & {\tilde{x}}_{22} & \begin{array}{cc} \ldots & {\tilde{x}}_{2n} \end{array}\\ \begin{array}{c} \vdots \\ {\tilde{x}}_{m1} \end{array} & \begin{array}{c} \vdots \\ {\tilde{x}}_{m2} \end{array} & \begin{array}{cc} \begin{array}{c} \ddots \\ \ldots \end{array} & \begin{array}{c} \vdots \\ {\tilde{x}}_{mn} \end{array} \end{array} \end{array}\right] \end{array},\;i=1,\;2,\;\ldots ,m;j=1,\;2,\;\ldots ,\;n$			(7)
	where:			
	*m*	–	number of alternatives	
	*n*	–	number of criteria/sub-criteria	
**Step 2.**	Normalization of decision matrix $\tilde{R}={\left[{\tilde{v}}_{ij}\right]}_{mxn}$			
	for stimulants:			
	${\tilde{r}}_{ij}=\left(\frac{l_{ij}}{u_{j}^{+}},\frac{m_{ij}}{u_{j}^{+}},\frac{u_{ij}}{u_{j}^{+}}\right),\;u_{j}^{+}=u_{ij}$			(8)
	for destimulants:			
	${\tilde{r}}_{ij}=\left(\frac{l_{j}^{-}}{u_{ij}},\frac{l_{j}^{-}}{m_{ij}},\frac{l_{j}^{-}}{l_{ij}}\right),\;l_{j}^{-}=l_{ij}$			(9)
**Step 3.**	Weighted normalized decision matrix where individual values are obtained by multiplying normalized fuzzy numbers by weight coefficients of criteria vector $\tilde{V}={\left[{\tilde{v}}_{ij}\right]}_{mxn}$			
	${\tilde{v}}_{ij}={\tilde{v}}_{ij}w_{ij}$			(10)
**Step 4.**	Determination of ordinates for FPIS ${\tilde{A}}^{+}$ (fuzzy positive ideal solution) and FNIS ${\tilde{A}}^{-}$ (fuzzy negative ideal solution) alternatives:			
	${\tilde{A}}^{+}=\left(\underset{i}{\max}\left({\tilde{v}}_{i1}\right),\;\underset{i}{\max}\left({\tilde{v}}_{i2}\right),\;\ldots ,\;\underset{i}{\max}\left({\tilde{v}}_{in}\right)\right)=\left({\tilde{v}}_{1}^{+},\;{\tilde{v}}_{2}^{+},\;\ldots ,{\tilde{v}}_{n}^{+}\right)$			(11)
	${\tilde{A}}^{-}=\left(\underset{i}{\min}\left({\tilde{v}}_{i1}\right),\;\underset{i}{\min}\left({\tilde{v}}_{i2}\right)\ldots ,\;\underset{i}{\min}\left({\tilde{v}}_{in}\right)\right)=\left({\tilde{v}}_{1}^{-},\;{\tilde{v}}_{2}^{-},\;\ldots ,{\tilde{v}}_{n}^{-}\right)$			(12)
**Step 5.**	Calculation of distances of each alternative from positive and negative ideal solution respectively:			
	$d_{i}^{+}=\overset{n}{\underset{j=1}{\sum }}d\left({\tilde{v}}_{ij},\;{\tilde{v}}_{j}^{+}\right)$			(13)
	$d_{i}^{-}=\overset{n}{\underset{j=1}{\sum }}d\left({\tilde{v}}_{ij},\;{\tilde{v}}_{j}^{-}\right)$			(14)
	$\mathrm{distance}\;\mathrm{between}\;\mathrm{two}\;\mathrm{triangular}\;\mathrm{fuzzy}\;\mathrm{numbers}\;{\tilde{x}}_{1}=\left(l_{1},\;m_{1},n_{1}\right)$$\mathrm{and}\;{\tilde{x}}_{2}=\left(l_{2},\;m_{2},n_{2}\right)$ is defined as follows:			
	$d\left({\tilde{x}}_{1},{\tilde{x}}_{2}\right)=\sqrt{\frac{1}{3}\left[{\left(l_{1}-l_{1}\right)}^{2}+{\left(m_{1}-m_{1}\right)}^{2}+{\left(u_{1}-u_{1}\right)}^{2}\right]}$			(15)
**Step 6.**	Calculation of the relative closeness of each alternative to the ideal solution:			
	$S_{i}=\frac{d_{i}^{-}}{d_{i}^{+}+d_{i}^{-}}$			(16)

When rating destimulants, the lower the value the higher the rating was given.

**Table 6:** Linguistic scales and corresponding scales used in EA FAHP and FuzzyTOPSIS methods, developed based on [56,58,61].

EA FAHP/FuzzyTOPSIS **		Corresponding Fuzzy Number	Reversed Fuzzy Number
EA FAHP	FuzzyTOPSIS		
Equivalence	Very low	(1, 1, 1)	(1, 1, 1)
	Low	(1, 2, 4) *	(1/4, 1/2, 1)
Minor dominance	Medium low	(1, 3, 5)	(1/5, 1/3, 1)
	Medium	(2, 4, 6) *	(1/6, 1/4, 1/2)
Strong dominance	Medium high	(3, 5, 7)	(1/7, 1/5, 1/3)
	High	(4, 6, 8) *	(1/8, 1/6, 1/4)
Very strong dominance	Very high	(5, 7, 9)	(1/9, 1/7, 1/5)
	Very very high	(6, 8, 9) *	(1/9, 1/8, 1/6)
Absolute dominance	Extremely high	(7, 9, 9)	(1/9, 1/9, 1/7)

* intermediate values (in case of EA FAHP method); ** column 4 shows a range of rating relevant for both EA FAHP and FuzzyTOPSIS methods.

**Table 7:** Decision matrix for FuzzyTOPSIS (ranks given as modal values).

Alternatives	Aggregated Weights of w_i_^E^ Criteria/Sub-Criteria Obtained Using EA FAHP Method											
	A1.1* 0.136	A1.2* 0.019	A2.1* 0.136	A2.2* 0.211	A2.3* 0.034	A2.4* 0.034	A2.5* 0.050	B 0.111	C1* 0.011	C2* 0.052	D1* 0.184	D2* 0.021
S1	1	5	1	1	1	1	1	2	9	1	1	9
S2	1	8	5	2	3	7	9	6	3	8	2	8
S3	2	9	3	3	3	6	9	4	2	6	4	6
S4	1	7	1	1	3	4	4	2	9	3	5	5
S5	2	6	2	2	1	9	9	3	5	7	3	7
S6	1	2	1	1	1	9	9	1	4	6	4	6
S7	1	9	9	3	4	8	8	8	5	6	4	6
S8	7	6	9	9	4	8	9	9	2	8	2	8
S9	7	1	6	7	6	7	9	7	1	8	2	8
S10	1	8	4	6	5	7	7	5	2	6	4	6
S11	9	9	9	8	8	9	9	8	3	8	2	8
S12	9	9	9	8	9	9	9	8	2	9	1	9

**Table 8:** Closeness coefficients (S_i_) of the alternatives (data were supplemented with intermediate values—distances d_i_^+^, d_i_^−^).

Closeness Variables	Alternatives											
	S01	S02	S03	S04	S05	S06	S07	S08	S09	S10	S11	S12
d_i_^+^	0.7324	0.4502	0.4394	0.5765	0.4953	0.5810	0.3246	0.1353	0.2394	0.3649	0.1068	0.1503
d_i_^−^	0.0489	0.3585	0.3806	0.2151	0.3240	0.1999	0.4756	0.6570	0.5743	0.4419	0.6810	0.6321
S_i_	0.0626	0.4433	0.4642	0.2717	0.3955	0.2560	0.5944	0.8293	0.7058	0.5477	0.8645	0.8079

## Data Availability

Data is contained within the article.
